# Effectiveness of external myofascial mobilisation in the management of male chronic pelvic pain of muscle spastic type: A retrospective study

**DOI:** 10.1080/2090598X.2021.1954414

**Published:** 2021-07-26

**Authors:** M. S Ajimsha, Laith Ahmad Ismail, Noora Al-Mudahka, Ahmad Majzoub

**Affiliations:** a Department of Physiotherapy, Hamad Medical Corporation, Doha, Qatar; b Department of Urology, Hamad Medical Corporation, Doha, Qatar; c Department of Urology, Weill Cornell Medicine – Qatar, Doha, Qatar

**Keywords:** Chronic prostatitis (CP), chronic pelvic pain syndrome (CPPS), pelvic floor physical therapy (PFPT), myofascial mobilisation, myofascial release, fascial connectivity

## Abstract

**Objective:**

To evaluate the outcome of men with muscle spastic chronic pelvic pain syndrome (CPPS) who underwent a comprehensive five-session fascial connectivity based external myofascial mobilisation (EMM) approach.

**Patients and methods:**

A retrospective chart review of patients who underwent EMM for CPPS at the Pelvic Pain Unit of Hamad Medical Corporation, Qatar between January 2019 and October 2020 was conducted. Patient’s symptoms were measured with the National Institutes of Health-Chronic Prostatitis Symptom Index (NIH-CPSI) scale and the numerical rating scale (NRS) before and after the completion of the sessions. The patients were given five EMM sessions as a ‘once-a-week’ programme.

**Results:**

A total of 31 patients who completed all the EMM sessions were included. The mean (range) age of patients was 38 (20–54) years. The mean (SD) NIH-CPSI score at initial evaluation was 29.41 (8.3) and decreased to 9.14 (3.45) after the fifth visit. All the patients in the study group had a reduction of >6 points in the NIH-CPSI score, indicating a robust treatment response. The NRS reading also revealed significant improvement in pain (*P* < 0.001).

**Conclusions:**

: An EMM approach based on fascial connectivity led to significant symptom improvement in all the studied patients. EMM may be an effective treatment option for muscle spastic type of CPPS. Future high-quality studies with control groups are needed to confirm the present findings. Durability and long-term results are yet to be determined.

**ABBREVIATIONS:**

CP/CPPS: chronic prostatitis/chronic pelvic pain syndrome; EMM: external myofascial mobilisation; EO: external oblique; FM: fascial manipulation; GMx: gluteus maximus; HAC: hip adductor complex; HMC: Hamad Medical Corporation; IO: internal oblique; LD: latissimus dorsi; MFR: myofascial release; MM: myofascial mobilisation; NIH-CPSI: National Institute of Health-Chronic Prostatitis Symptom Index; NRS: numerical rating scale; PFPT: pelvic floor physical therapy; QoL, quality of life; TLF: thoracolumbar fascia; UPOINT: urinary (U), psychosocial (P), organ-specific (O), infection (I), neurological/systemic (N) and tenderness of pelvic floor skeletal muscles (T)

## Introduction

Chronic prostatitis/chronic pelvic pain syndrome (CP/CPPS) is a devastating medical condition affecting men of all ages and constitute ~10% of outpatient department visits to Urologists worldwide, with significant impact on quality of life and financial burden [1,2]. In fact, ~25% of men experience loss of work and ~50% show a reduction in leisure time activities at some point due to CPPS [3]. CPPS is characterised by symptoms lasting ≥3 months during the past 6 months, in the absence of a bacterial UTI [4]. The creation of a syndrome-based diagnostic classification (UPOINT system; UPOINT phenotypes: urinary [U], psychosocial [P], organ-specific [O], infection [I], neurological/systemic [N], and tenderness of pelvic floor skeletal muscles [T]) by the National Institutes of Health (NIH) [4] has allowed for the development of a symptom-focussed treatment approach. Moreover, the European Association of Urology (EAU) guidelines on CPPS recommends a multimodal approach to achieve the best outcomes [5].

There are encouraging studies on the effectiveness of non-pharmacological approaches, including pelvic floor physical therapy (PFPT) in the management of CPPS [6–8]. PFPT encompasses a wide range of treatments that physicians are often not aware of. These include myofascial release [9,10], massage [11], exercise and stretching [12], biofeedback [13], electrotherapy [14], and neuromodulation [15]. Up to 85% of men with CPPS may have pelvic floor tenderness [16] and these areas of tenderness reproduce the patient’s pain with palpation in many cases [17]. For these patients, the first line of care is usually PFPT. Even in patients who have failed other treatments, it has been shown that PFPT improves symptoms in as many as 72% of these patients [18].

Pelvic myofascial mobilisation (MM) refers to the manual therapy of the fascia in and around the pelvis. The MM application can be external or internal. The external MM (EMM) is the application of manual therapy based on the myofascial connectivity [19] and myofascial force transmission [20]. Here the fascia around the pelvis will be examined and mobilised for myofascial dysfunction in a traceable pattern. The most common MM procedures are myofascial release (MFR) and fascial manipulation (FM). The MFR involves the application of a variable load and long duration stretch to the myofascial complex intended to restore optimal length, decrease pain, and improve function [21]. In FM, the fascia will be manipulated through predefined myofascial units, that, when treated appropriately, are believed to restore tensional balance [22]. Evidence is accumulating regarding the administration of internal MM [10], but this procedure is less comfortable and culturally sensitive for many patients as this involves per rectal application of the MM.

The Pelvic Pain Unit developed by the Urology Department at Hamad Medical Corporation is the first of its kind in the region to provide a multidisciplinary evidence-based treatment approach to the men with CPPS. Myofascial physical therapy is added as an integral part of this service where the patient will be referred for PFPT based on the UPOINT classification. The major referral criterion for PFPT is a positive ‘T’ (tenderness) phenotype in the UPOINT system.

External MM is being used to treat patients with CPPS anecdotally, but there is an insufficiency of supporting evidence. The present study aimed to determine the effectiveness of an external fascia oriented soft tissue mobilisation on pain and symptom severity in male patients with chronic pelvic pain with muscle tenderness phenotype through a retrospective data review.

## Patients and methods

### Study design

This study was carried out in the Urology Department in the Ambulatory Care Centre of Hamad Medical Corporation (HMC), Qatar. The HMC Research Ethics Board reviewed the study and raised no objection from an ethical perspective with a waiver of signed informed consent. A retrospective chart audit was performed by utilising the physician and physiotherapists evaluation and re-evaluation forms to extract the patient’s demographic and clinical details. Data were extracted from the Cerner Millenium for men aged >18 years who were referred by a urology pelvic pain physician for PFPT from January 2019 to October 2020 with a diagnosis of muscle spastic CPPS after examination, based on the UPOINT classification system. Men who had clearly identifiable causes of pelvic pain, such as prior surgery, chronic infection, trauma, prostatitis and epididymitis, were excluded. Men with concurrent urinary incontinence or prostatectomy were also excluded. Using the UPOINT phenotype classification system, all patients reported positive phenotype for pelvic floor tenderness. All the patients were evaluated and treated by the same myofascial physiotherapists experienced in the EMM. Symptom severity was measured with the NIH Chronic Prostatitis Symptom Index (CPSI) and numerical rating scale (NRS). We identified 31 patients who met the inclusion criteria and who had completed all sessions after receiving information about the CPPS and the EMM therapy programme.

### Procedures

**Figure 1.**
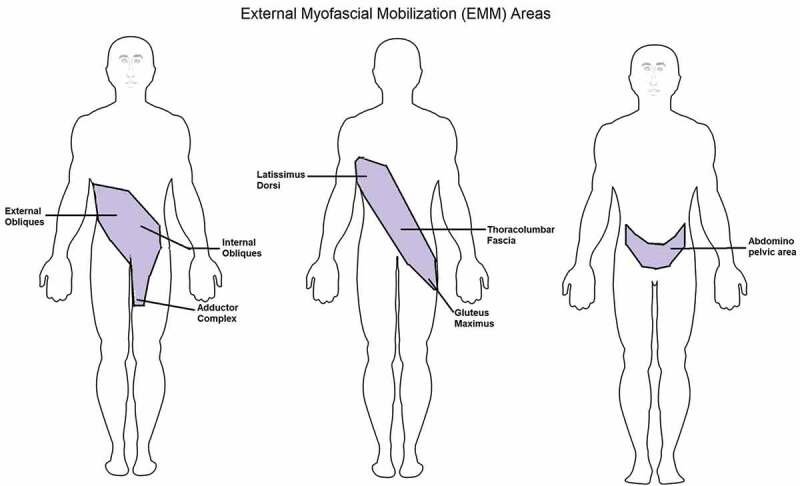
EMM treatment areas

The EMM therapy for CPPS mobilised the fascia around the lumbopelvic area by following the fascial connectivity of the trunk’s oblique chain system [23] and the abdomino-pelvic viscera [24] (Figure 1). The EMM treatment area extended from ipsilateral latissimus dorsi (LD), ipsilateral thoracolumbar fascia (TLF) and contralateral gluteus maximus (GMx) posteriorly, and ipsilateral external oblique (EO) and contralateral internal oblique (IO) and hip adductor complex (HAC) anteriorly. After the completion of the initial examination and possible exclusion based on contraindications, patients suitable for EMM therapy were given five therapy sessions once a week, with each session having an average duration of 30 min. The techniques were administered by physiotherapists specialised in fascial mobilisation with a median experience of 15 years. The EMM is a ‘hands-on’ therapy that is administered with bare hands and fascial guns to mobilise the fascial restrictions in the designated areas of dysfunction. The sessions included the EMM of: (1) right EO, left IO and HAC; (2) left EO, right IO and HAC; (3) right LD, TLF and left GMx; (4) left LD, TLF and right GMx; (5) EMM of the abdomino-pelvic viscera. The detail of the treatment sessions is beyond the scope of this review.

### Data collection

Demographic and clinical data were extracted from patients’ records. Patients’ progress was measured using the NIH-CPSI and NRS scales, administered at the initial evaluation and 1 week after the fifth session. The NIH-CPSI has a total score range from 0 to 43, with three subscales addressing pain (score range 0–21), urinary symptoms (score range 0–10), and quality of life (QoL) (score range 0–12) [25]. Higher scores reflect worse symptoms. A 6-point reduction in the CPSI score is regarded as a clinically meaningful improvement of symptoms [6]. The NRS scale was used to track the patient’s subjective and general pain complaints.

### Statistical analysis

Frequencies (%) and means (SDs) were used to present categorical and numerical data, respectively. Descriptive statistics were used to measure patient progress. Patient characteristics, including age, duration and severity of symptoms, phenotype distributions and adverse events were assessed at different time intervals. The mean pre and post CPSI and NRS scores were compared using the paired *t*-test. The chi-squared test was used to assess changes in symptom severity after the intervention. All statistical analyses were done using the Windows version of the Statistical Package for the Social Sciences (SPSS®), version 25 (IBM Corp., Armonk, NY, USA).

## Results

### Characteristics of the study population

Patients demographic profile and baseline data are reported in Table 1. The mean (range) age of patients was 38 (20–54) years and the symptom duration before the treatment was 42 (13–103) months. Presence of associated symptoms reported by the patients was documented as ‘Yes/No’, with all patients (100%) reporting pain, 97% patients reporting pelvic symptoms, besides urinary (52%) and sexual (26%) symptoms. Most patients (45%) reported at least two associated symptoms and 39% reported at least three. Only 13% had all the associated symptoms (Table 2).

**Table 1.** Demographic and baseline clinical data of patients

Characteristic	Value
Number of patients	31
Age, years, mean (SD; range)	38.41 (7.43; 20–54)
Duration of the condition (months), mean (SD; range)	42.57 (27.44; 13–103) Median 33
Presence of associated Symptoms, *n* (%)	
Pain	31 (100)
Pelvic	30 (97)
Urinary	16 (52)
Sexual	8 (26)
Prevalence of UPOINT phenotypes, *n* (%)	
Tenderness of skeletal muscles	31 (100)
Urinary	17(54.8)
Psychological	6 (19.4)
Organ specific	21 (67.7)
Infection	2 (6.5)
Neurological/system specific	5 (16.1)
Baseline NIH-CPSI score and sub-scores, mean (SD; range)	
Total score	29.41 (8.30; 20–37)
Domain 1: Pain	16.32 (3.62; 11–17)
Domain 2: Urinary symptoms	5.78 (1.96; 1–9)
Domain 3: QoL	7.31 (3.24; 1–11)
Pain reported (NRS), mean (SD; range)	6.18 (1.24; 4–8)

**Table 2.** Reported symptoms and UPOINT phenotypes at baseline

Symptoms reported at baseline, *n* (%)		Positive UPOINT phenotypes reported at baseline, *n* (%)	
Reported Only 1	1 (3)	Reported Only 1	1 (3)
Reported 2	14 (45)	Reported 2	11 (36)
Reported 3	12 (39)	Reported 3	14 (45)
Reported All 4	4 (13)	Reported 4	5 (16)
Symptoms: Pain, Pelvic, Urinary, Sexual		Reported 5	0
		Reported All 6	0
		UPOINT phenotypes: urinary (U), psychosocial (P), organ-specific (O), infection (I), neurological/systemic (N) and tenderness of pelvic floor skeletal muscles (T)	

**Figure 2.**
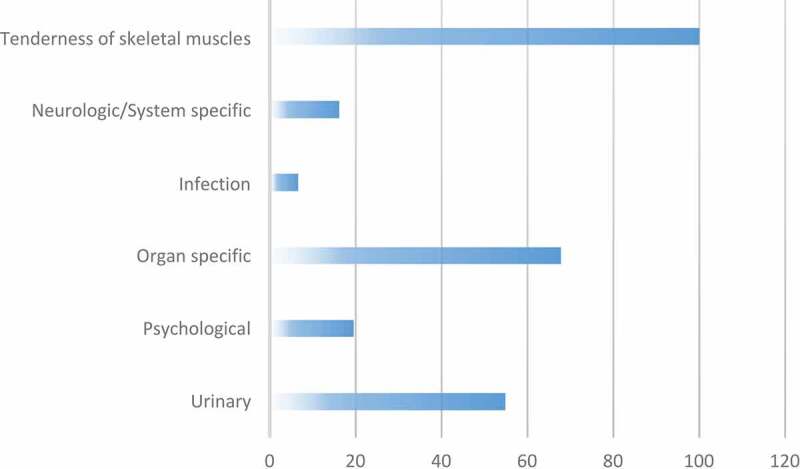
Prevalence of UPOINT phenotypes in patients (%)

Assessment of the UPOINT phenotype revealed positive ‘tenderness’ of skeletal muscle phenotype in all the patients. The data also revealed 68% positive phenotype for organ specificity followed by the urinary (55%) phenotypes. Only 20% and less reported other phenotypes (neurological/system specific, psychological and infection) (Figure 2). Studying the frequency of these phenotypes (Table 2) showed 45% patients with three positive phenotypes, 36% with two positive phenotypes, 16% reported at least four and only 3% reported a single phenotype. None of the patients had reported five or more phenotypes in this group, i.e. all patients had at least two positive phenotypes and most had three.

The mean (SD; range) total NIH-CPSI score (Table 1) before treatment was 29.41 (8.3; 20–37) for a total maximum score of 43. The mean (SD) NIH-CPSI of pain, urinary and QoL sub-scores before treatment was 16.32 (3.62), 5.78 (1.96), and 7.31 (3.24), respectively.

### Impact of intervention

**Figure 3.**
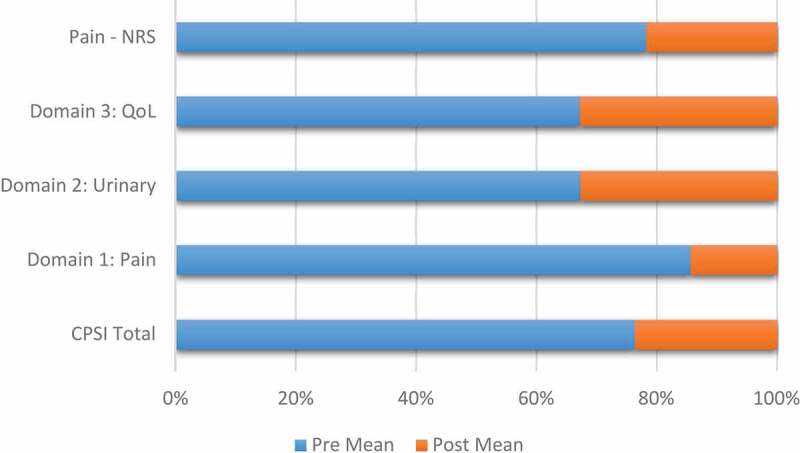
Change in the CPSI and NRS scales over time

To identify the effect of treatment, we compared the NIH-CPSI total score, sub-score, and pain score (NRS) before (pre) and after intervention (post). The results indicated a significant improvement in all NIH-CPSI scores as well as pain scale (Table 3, Figure 3). The average improvement in the total score was by 20 points with ~50% of that change primarily due to improvement in the sub-score of pain, with an average improvement of 13 points. The proportion of responders, defined as participants who had a ≥ 50% reduction in their pelvic symptoms after the fifth session was 94%.

**Table 3.** Change in NIH-CPSI total score, sub-scores and pain (NRS) before and after EMM

NIH-CPSI score	Before EMM, mean (SD)	After EMM, mean (SD)	Difference, mean (SD)	95% CI	*P*
Total	29.41 (8.30)	9.14 (3.45)	20.28 (5.63)	(19.43–21.43)	<0.001
Domain 1: Pain	16.32 (3.62)	2.88 (1.98)	13.44 (3.12)	(12.42–13.94)	<0.001
Domain 2: Urinary	5.78 (1.96)	2.81 (1.31)	2.97 (1.83)	(2.42–3.64)	<0.001
Domain 3: QoL	7.31 (3.24)	3.57 (2.32)	3.74 (2.15)	(3.17–4.30)	<0.001
Pain – NRS	6.18 (1.24)	1.72 (1.54)	4.46 (2.01)	(4.12–5.02)	<0.001

**Figure 4.**
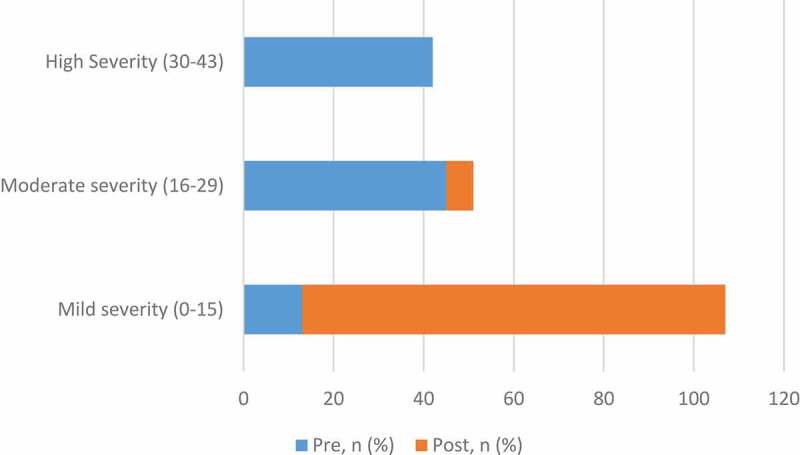
Change in symptom severity before and after EMM

We further analysed the treatment effectiveness through determining change in symptom severity. Patients’ symptom severity was classified as mild (0–15), moderate (16–29) or severe (>29) based on their total score (Table 4). Before the implementation of treatment, most patients had severe (42%) or moderate symptoms (45%). However, after treatment results demonstrated a significant reversal of trend, where 94% of the patients reported only mild symptoms, 6% reported moderate symptoms, and none reported severe symptoms (Figure 4).

**Table 4.** Change in symptom severity as per NIH-CPSI total score

NIH-CPSI score severity	Before EMM, *n* (%)	After EMM, *n* (%)
Mild severity (0–15)	4 (13)	29 (94)
Moderate severity (16–29)	14 (45)	2 (6)
High Severity (30–43)	13 (42)	0 (0)

Application of the EMM therapy resulted in a few adverse effects such as pain, dysuria, feverishness and skin discoloration in the area of MM application. All the adverse effects recorded were temporary and were reported mainly during the initial sessions of the therapy. None of the patients reported any adverse effects when assessed after the fifth sessions of the therapy (Table 5).

**Table 5.** Adverse effects reported

Adverse effects reported	After EMM session, %				
	One	Two	Three	Four	Five
Pain	36	19	10	0	0
Dysuria	16	7	0	0	0
Feverishness	10	3	0	0	0
Skin discoloration	26	13	7	0	0

## Discussion

A comprehensive five session EMM programme based on fascial connectivity principle improved symptoms when measured with CPSI and NRS scales in men with muscle spastic CPPS. An EMM approach led to significant symptom improvement in all the present studied patients. All the patients reported a significant improvement in symptoms as measured by a minimum 6-point change in their CPSI score. No participants had worsening of symptoms. Most the patients (94%) had a ≥ 50% reduction in their pelvic symptoms after the fifth session. Moreover, the five session EMM therapy resulted in a significant reduction of symptom severity in 94% of the patients. The treatment was safe, and we observed only minimal immediate adverse effects that were of a short duration. This indicates that EMM is a reasonable and cost-effective adjunct for the treatment of men with muscle spastic CPPS and is unlikely to be harmful or worsen symptoms.

Chronic pelvic pain syndrome remains a conundrum for physicians and patients because of the heterogeneity of the symptoms [26]. Monotherapy with medication or psychotherapy is often ineffective and can lead to frustration for both the patient and the practitioner [26]. For most practitioners, medical therapy directed at discrete symptoms is the first-line option. Antibiotics are frequently prescribed, even if evidence of infection is lacking. Widely available medications, such as α-blockers, anticholinergics and anti-inflammatories can be helpful in some cases [27]. A 2018 meta-analysis [6] and 2019 systematic reviews [7,8] that evaluated the effectiveness of non-medical approaches, including manual therapy for CP/CPPS concluded that treating CPPS by means of non-pharmacological and physical therapies including myofascial methods can significantly improve the outcomes. Men with CPPS often have pelvic floor tension and tenderness [16]. Treating this pelvic floor spasm can yield significant improvement in patients’ symptoms. Most of the studies conducted to date on the myofascial concept utilised a multitude of approaches of internal and external trigger points with the goal or relieving muscle tension and pain [6,10,11,28,29]. The techniques utilised include direct pressure, proprioceptive neuromuscular techniques, deep tissue mobilisation, myofascial and trigger point mobilisation procedures [8]. In a large study, involving 138 men with refractory CPPS, 72% reported substantial to moderate improvement as measured by the NIH-CPSI score [26]. Another study with 106 men with refractory CPPS showed improvement in pain by visual analogue scale with the use of a specially designed wand for releasing internal trigger points (7.5 to 4) [10]. The present study utilised a scientific rationale developed based on the recent understanding of the myofascia such as its global connectivity [19], ability to transfer myofascial force [20], fascial layer sliding [22], shock absorbing capacity, and its sensory and autonomic innervation properties [30].

The EMM therapy for CPPS mobilise the fascia around the lumbopelvic area. Recent fascial analysis studies found that fascia is actively taking part in force transmission and is richly innervated by autonomic fibres [30], with total innervation density (afferent + efferent) more than that of the muscles [31]. The fascia is a continuous structure, and any dysfunction of the body will in turn cause the dysfunction of the fascia by affecting its sliding properties, force transmission capacities, and change in the fascial innervation types and densities (pathological alteration) [31]. MM improves fascial sliding [22], myofascial force transmission [20] and fascial plasticity, which in turn has an influence on the fascial connectivity and mobility locally and remotely [32]. EMM requires training and experience in fascial mobilisation and an in-depth knowledge about the condition and effects of therapies on it [33].

The present retrospective analysis of the data found that the EMM based on the fascial connectivity approach led to a significant improvement in symptoms in all the patients studied. EMM may be an effective treatment adjunct for the muscle spastic type of CPPS. Further high-quality studies are required to verify these findings. Sustainability and long-term outcomes have not yet been determined.

To the best of our knowledge, the present study is the first of its kind to assess a comprehensive five-session EMM therapy programme. As shown, the EMM approach led to a significant reduction (69%) in the CPSI scores, with all patients reporting a robust response. Item analysis of the CPSI scale revealed that most of the improvement was in the pain symptoms, with almost 82% reduction from the pre-test pain score. The general pain assessment with a NRS scale substantiated the above finding, with a 72% reduction of the pain after therapy. Interestingly, the urinary and QoL components also showed a minimum of 50% change in scores. It is worth noting that the patient’s data extracted were from patients with a positive tenderness phenotype and the reduced presentation of psychological, infection and organ-specific phenotypes along with relatively young age of the patients might have an influence on the results. Future studies should analyse the influence of such covariables. More importantly, no patients had worsened symptoms or serious adverse events during the therapy. Strengths of the present study are that comprises patient data of the positive tenderness phenotype, a relatively young patient group, and homogeneity of therapy provided. All patients received similar therapies for a fixed duration and frequency. The major limitations of the present study are related to its retrospective design and small sample size. A sample size with a relatively young population with highly controlled inclusion criteria can induce a super realisation bias on the results. The early administration of EMM therapy regardless of the medical therapy received might have influenced the outcome. The present study is without a follow-up to ascertain the sustainability of the result. Additionally, retrospective analysis limits the controlling of the confounders that can influence the study outcomes and the placebo effects.

## Conclusion

Chronic pelvic pain syndrome is a common but debilitating chronic condition that affects men and women, and results in significant economic burdens and healthcare costs. The anecdotal evidence of the effectiveness of EMM for CPPS is receiving a great deal of attention and the present study provides proof of concept for its effectiveness. The EMM may be an effective therapeutic supplement for the muscle spastic type of CPPS. The sustainability of long-term effects and outcomes remains to be determined. Future prospective and blinded, randomised studies with adequate sample size and long-term follow-up are needed to ascertain the above findings and to predict characteristics of men who would respond to the EMM therapy.
